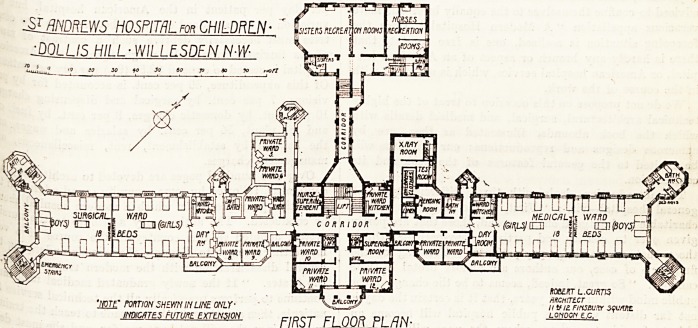# St. Andrew's Hospital, Dollis Hill, Willesden

**Published:** 1913-07-19

**Authors:** 


### HOSPITAL ARCHITECTURE AND CONSTRUCTION.

### St. Andrew's Hospital, Dollis Hill, Willesden.

This new hospital has been erected specially for Roman Catholic patients. It stands on the crest of Dollis Hill on a site of seven acres, and has an almost ideal situation for hospital purposes. The view to the south is over the vale of Cricklewood, to the west the Weald of Harrow, to the north over the valley of the Brent, and to the east towards Hampstead Heath and Golder's Green.

GROUND FLOOR PLAN·

The building as planned comprises a long front block facing south-east, with an extension at the back connected to the main front by a corridor. Only the north-east wing and the centre portion of the main block have been erected, together with the whole of the projecting block at the back.

The main entrance is in the centre of the building, the vestibule having on one side a porter's office, and on the other side a small cloak room gives access to a large hall, off which are waiting and reception rooms, secretary's office, and superior's room. At the back of the hall a corridor runs right and left, and communicates with the north-east wing on the one side, and will give access to the south-west wing when it is built.

At the back of this block is the main staircase, in the well of which is an electric lift for passengers, on either side being medical officers' common room and receiving room. The north-east wing contains a large ward of eighteen beds, two private wards of one bed each, a small day room, ward kitchen, bathroom for the private wards, nurses' room, linen room, a testing room, and the dispensing room. The angles of the ward are projected out: on one side a bathroom and other sanitary offices are placed; on the other, fire escape stairs; and on the south-east side of the ward is a broad terrace on to which the patients can be wheeled.

The sink rooms provided both for the large ward and the private wards are far too small; and the bathrooms for the wards would have been very much better placed so that they could have been entered immediately from the ward itself, instead of through the cut-off passage of the sanitary block.

The first floor is very similar to the ground floor, except that over the entrance hall and adjacent rooms are three more private wards, giving five private wards in all on this floor; and for these five private wards there is a separate ward kitchen. The $x$-ray room, with a dark room, takes the place of the dispensary on the floor below.

On the second floor the front portion of the centre block is entirely occupied by the chapel. Over the $x$-ray room is the operation room, the anæsthetic room, sterilising room, and robing room, and lavatories for the staff are placed in the projecting sanitary tower, which serves the private wards below. There is also a large cupboard for dressings. The sterilising room looks very small for the purpose, and there is no indication of where the washing-up is done. The space in the sterilising room is further restricted by the fact that it has to form the passage for exit of patients after operations.

The ward wing on this floor is occupied by bedrooms for sisters and servants, with bathrooms and lavatories. Part of the back block contains isolation wards for four beds, with a ward kitchen, a nurses' bathroom and changing room with a separate exit, and four nurses' bedrooms approached by a staircase from the floor below. These bedrooms are cut off by a brick wall from the isolation wards. The entrance to the isolation wards is by way of the flat roof over the corridor below. In the future block these isolation wards will be added to by the addition of three more beds in the block projecting to the north-west attached to the south-west wing, and the rest of the wing will be occupied by accommodation for sisters, with a sisters' sick room and a ward kitchen. A small room leading out of the sisters' sick room faces a tribune looking into the chapel.

When complete the hospital will give accommodation for 100 beds. The work has been carried out from designs and under the superintendence of Mr. Robert L. Curtis, 11 and 12 Finsbury Circus, London, E.C.

## Figures and Tables

**Figure f1:**
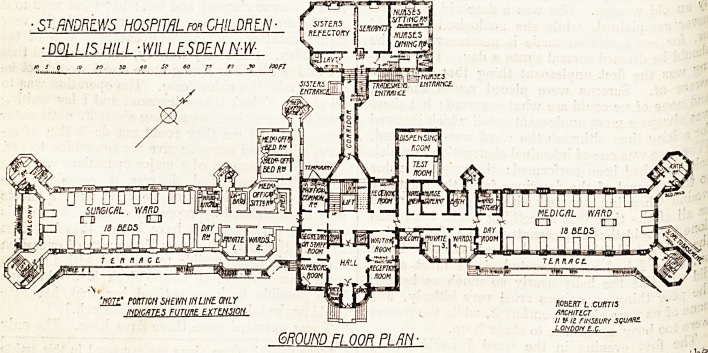


**Figure f2:**